# Oral vitamin C supplementation to patients with myeloid cancer on azacitidine treatment: Normalization of plasma vitamin C induces epigenetic changes

**DOI:** 10.1186/s13148-019-0739-5

**Published:** 2019-10-17

**Authors:** Linn Gillberg, Andreas D. Ørskov, Ammar Nasif, Hitoshi Ohtani, Zachary Madaj, Jakob W. Hansen, Nicolas Rapin, Johanne B. Mogensen, Minmin Liu, Inge H. Dufva, Jens Lykkesfeldt, Petra Hajkova, Peter A. Jones, Kirsten Grønbæk

**Affiliations:** 1 Department of Haematology, Rigshospitalet, Copenhagen Biocenter, Building 2, 3rd floor, Ole Maaløes Vej 5, DK-2200 Copenhagen, Denmark; 2 Biotech Research and Innovation Centre (BRIC), Faculty of Health and Medical Sciences, University of Copenhagen, Copenhagen, Denmark; 3 MRC London Institute of Medical Sciences (LMS), Imperial College, London, UK; 4 Van Andel Research Institute, Grand Rapids, MI USA; 5 The Danish Stem Cell Center (Danstem), Faculty of Health and Medical Sciences, University of Copenhagen, Copenhagen, Denmark; 6 Department of Haematology, Herlev University Hospital, Copenhagen, Denmark; 7 Department of Veterinary and Animal Sciences, Faculty of Health and Medical Sciences, University of Copenhagen, Frederiksberg, Denmark

**Keywords:** Vitamin C, Hydroxymethylcytosine, Myeloid cancer, Azacitidine, Epigenetics

## Abstract

**Background:**

Patients with haematological malignancies are often vitamin C deficient, and vitamin C is essential for the TET-induced conversion of 5-methylcytosine (5mC) to 5-hydroxymethylcytosine (5hmC), the first step in active DNA demethylation. Here, we investigate whether oral vitamin C supplementation can correct vitamin C deficiency and affect the 5hmC/5mC ratio in patients with myeloid cancers treated with DNA methyltransferase inhibitors (DNMTis).

**Results:**

We conducted a randomized, double-blinded, placebo-controlled pilot trial (NCT02877277) in Danish patients with myeloid cancers performed during 3 cycles of DNMTi-treatment (5-azacytidine, 100 mg/m^2^/d for 5 days in 28-day cycles) supplemented by oral dose of 500 mg vitamin C (*n* = 10) or placebo (*n* = 10) daily during the last 2 cycles. Fourteen patients (70%) were deficient in plasma vitamin C (< 23 μM) and four of the remaining six patients were taking vitamin supplements at inclusion. Global DNA methylation was significantly higher in patients with severe vitamin C deficiency (< 11.4 μM; 4.997 vs 4.656% 5mC relative to deoxyguanosine, 95% CI [0.126, 0.556], *P* = 0.004). Oral supplementation restored plasma vitamin C levels to the normal range in all patients in the vitamin C arm (mean increase 34.85 ± 7.94 μM, *P* = 0.0004). We show for the first time that global 5hmC/5mC levels were significantly increased in mononuclear myeloid cells from patients receiving oral vitamin C compared to placebo (0.037% vs − 0.029%, 95% CI [− 0.129, − 0.003], *P* = 0.041).

**Conclusions:**

Normalization of plasma vitamin C by oral supplementation leads to an increase in the 5hmC/5mC ratio compared to placebo-treated patients and may enhance the biological effects of DNMTis. The clinical efficacy of oral vitamin C supplementation to DNMTis should be investigated in a large randomized, placebo-controlled clinical trial.

**Trial registration:**

ClinicalTrials.gov, NCT02877277. Registered on 9 August 2016, retrospectively registered.

## Introduction

Our understanding of the aetiology of haematological malignancies has undergone a revolution with the discovery of a high frequency of mutations in epigenetic regulators, particularly those involved in the modification of cytosine. Premalignant clonal haematopoiesis and malignant myeloid disorders often have mutations in the *DNMT3A* or *TET2* genes [1–3] encoding enzymes that apply methyl groups to cytosine or oxidize 5-methylcytosine (5mC) to 5-hydroxymethylcytosine (5hmC), respectively [4–6]. In addition, extensive DNA methylation changes have been demonstrated as a hallmark of myeloid malignancies [7].

Here, we have focused on vitamin C (ascorbic acid), which serves as a cofactor for TET and Jumonji enzymes via enhanced recycling of reduced iron [8]. Recent reports from our group and others have shown that patients with haematological malignancies are often severely deficient in plasma vitamin C, for unknown reasons [6, 9–12]. Also, our studies in tissue culture have shown that the restoration of vitamin C to normal concentrations can potentiate the effects of DNA methyltransferase inhibitors (DNMTis) [11]. Today, DNMTis are the only drugs that prolong the overall survival in patients with higher-risk myelodysplastic syndrome (MDS) [13]. The mechanism of action of DNMTis has long been investigated; however, recent studies by us and others suggest that an important mechanism is via upregulation of transposable elements including endogenous retroviruses (ERVs) that may form double-stranded RNA which can be sensed by viral recognition receptors and induce viral defence pathways, so-called viral mimicry [14, 15].

We previously observed the synergistic inhibition of cancer cell proliferation by combining DNMTis at various concentrations with vitamin C at physiological levels in tissue culture [11]. The synergy between vitamin C and DNMTis is likely due to accentuated DNA demethylation by the combined effect of passive inhibition of DNA methylation by DNMTis, and vitamin C-stimulated production of 5hmC by the TET enzymes, which together may cause increased expression of ERVs and viral defence genes [11]. Given that more than 80% of patients in Denmark with haematological malignancies have very low concentrations of vitamin C in their plasma [11], we have conducted a randomized clinical pilot trial (NCT02877277) to determine whether physiological vitamin C levels could be re-established by daily oral intake of 500 mg of vitamin C in patients with myeloid cancers treated with DNMTis. We also investigated whether the vitamin C supplement influenced the global levels of 5hmC relative to 5mC and the expression of viral defence genes in the patients’ mononuclear myeloid cells.

## Results

### Plasma vitamin C deficiency in patients with myeloid cancers

**Fig. 1:**
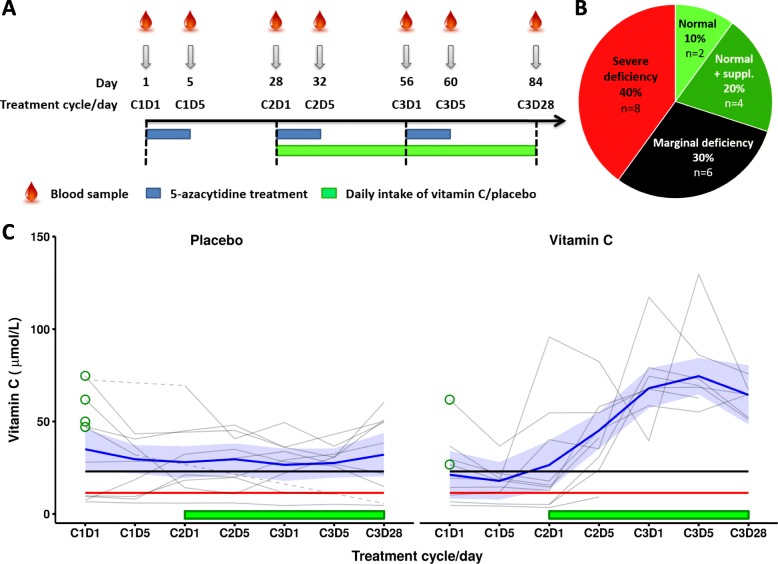
Study design and vitamin C levels at baseline and after patients had been randomized to 500 mg vitamin C or placebo. **a** Study design. Days 1, 5, and 28: before vitamin C/placebo exposure. Day 32: after short-term vitamin C/placebo exposure. Days 56, 60, and 84: after longer-term vitamin C/placebo exposure. **b** Plasma vitamin C status at baseline. Normal + suppl.: patients who had been taking vitamin C/multivitamin supplement at the time of inclusion. **c** Plasma vitamin C concentration at baseline and after randomization to vitamin C (500 mg) or placebo. Red line represents the threshold for severe vitamin C deficiency (11.4 μM) [16]. Black line represents the threshold for vitamin C deficiency (23 μM). Patients who had been taking vitamin C supplements at the time of study inclusion are marked using green circles. Time-periods of daily intake of vitamin C or placebo are highlighted using green boxes. Dotted lines indicate timepoints with missing plasma vitamin C levels. Data presented as spline mean (blue line) with standard error ribbons for cycles; C cycle, D day in cycle.

We enrolled 20 Danish patients with myeloid cancers (9 MDS, 7 acute myeloid leukaemia (AML), and 4 chronic myelomonocytic leukaemia (CMML) patients) who were undergoing treatment with 5-azacytidine and followed them for a total of three treatment cycles after enrolment (Fig. 1a). Any previous intake of vitamin C supplements by the participants was discontinued at the time of inclusion. Patient characteristics and the mutational status of DNA methylation regulators at baseline are given in Table 1 and in Additional file 1: Table S1. Seven patients had *TET2* mutations, five had *DNMT3A* mutations, and two had mutations in *IDH2* (Table 1 and Additional file 1: Table S2). At baseline, 14 patients were vitamin C deficient (< 23 μM) and eight of these were severely deficient (< 11.4 μM); the remaining six patients had plasma vitamin C above the deficiency threshold of 23 μM [16] (Fig. 1b). Notably, four of the latter six patients had been taking vitamin C dietary supplements regularly prior to study inclusion. Plasma vitamin C at baseline was not significantly different in patients with or without mutations in epigenetic regulators (Additional file 1: Fig. S1).

**Table 1:** Baseline characteristics of patients randomized for vitamin C (500 mg) or placebo supplement

Patients	All	Vitamin C	Placebo
Number	20	10	10
Sex (men/women)	14/6	9/1	5/5
Age at inclusion (years)	73 (57–84)	76 (70–84)	70 (57–84)
Diagnosis			
MDS	9	5	4
AML	7	2	5
CMML	4	3	1
DNMTi treatment before inclusion (cycles)			
0	11	5	6
1	3	1	2
2	3	2	1
3	1	1	0
4–7	2	1	1
IPSS-R score	5 (3.5–6.5)	4.5 (4–5.5)	5.5 (3.5–6.5)
Haemoglobin, g/dL	9.6 (7.1–14)	9.4 (7.1–14)	9.9 (7.6–12)
No. of blood transfusions in the study period	7 (0–33)	10 (0–33)	1 (0–18)
*TET2* mutation, % of all (yes/no)	35% (7/13)	40% (4/6)	30% (3/7)
*DNMT3A* mutation, % of all (yes/no)	25% (5/15)	30% (3/7)	20% (2/8)
*IDH2* mutation, % of all (yes/no)	10% (2/18)	20% (2/8)	0% (0/10)
Vitamin C, μmol/L^a^	21 (5–73)	14 (5–37)	27 (7–73)
Iron, μmol/L^a^	17 (2–33)	18 (2–33)	16 (5–32)
Transferrin, mg/dL^a^	184 (116–257)	169 (116–237)	199 (148–257)
Ferritin level, μg/L^a^	1624 (95–6244)	1993 (444–6244)	1254 (95–3632)

Data are given as mean (range) except for IPSS-R score and blood transfusions, which are given as median (range). There were no statistically significant differences between groups. No patients had mutations in *IDH1*

*MDS* myelodysplastic syndrome, *AML* acute myeloid leukaemia, *CMML* chronic myelomonocytic leukaemia, *DNMTi* DNA methyltransferase inhibitor, *IPSS-R* Revised International Prognostic Scoring System (at the date of diagnosis for MDS and AML patients with < 30% bone marrow blasts)

^a^Plasma concentration at baseline

### Significant difference in plasma vitamin C levels after supplementation

Plasma vitamin C quickly dropped in patients who stopped their previous vitamin supplements (Fig. 1c). From day 1 of treatment cycle 2 (C2D1) in the trial regimen, the patients were randomized in a 1:1 ratio to receive oral doses of vitamin C (500 mg/d) or placebo. On day 1 and 5 of the two following treatment cycles and by the end of the third treatment cycle, blood samples were drawn immediately before 5-azacytidine was administered (Fig. 1a). There was a clear improvement of plasma vitamin C in those patients who received a daily oral dose of 500 mg of vitamin C. After only 4 days of supplementation, vitamin C levels were significantly increased (mean difference ± SE, 36.31 ± 9.67 μM; *P* = 0.0011; Additional file 1: Table S3). Plasma vitamin C remained high during the third treatment cycle in the patients randomized to vitamin C (34.85 ± 7.94 μM, *P* = 0.0004, relative to before supplementation), but it was not significantly different from short-term (4-day) supplementation. Plasma vitamin C on day 28 (C3D28) was lower than on days 1 and 5 of treatment cycle 3 (C3D1 and C3D5), but this difference was not statistically significant. Patients in the placebo arm showed minor changes in plasma vitamin C levels as a function of time, which were not statistically significant (Fig. 1c and Additional file 1: Table S3). Vitamin C levels were similar between groups at baseline, but significantly different after short- (mean difference ± SE, 36.07 ± 10.69 μM; *P* = 0.0016) and longer-term (32.78 ± 9.08 μM, *P* = 0.0013) supplementation (Additional file 1: Table S3). There were no significant associations between plasma levels of vitamin C and ferritin, transferrin, or iron (Additional file 1: Table S4), and vitamin C concentrations at baseline were not associated with WHO diagnosis, MDS prognostic risk category (IPSS-R score), blast percentage, haemoglobin level, or the number of blood transfusions, in this heterogeneous patient cohort (Additional file 1: Table S5A). Plasma levels of ferritin varied considerably between patients and were above the normal range in patients receiving blood transfusions, but the levels of ferritin, transferrin, and iron for any individual patient did not change significantly during the trial regimen (Additional file 1: Fig. S2).

### Vitamin C supplement increases 5hmC/5mC relative to placebo

**Fig. 2:**
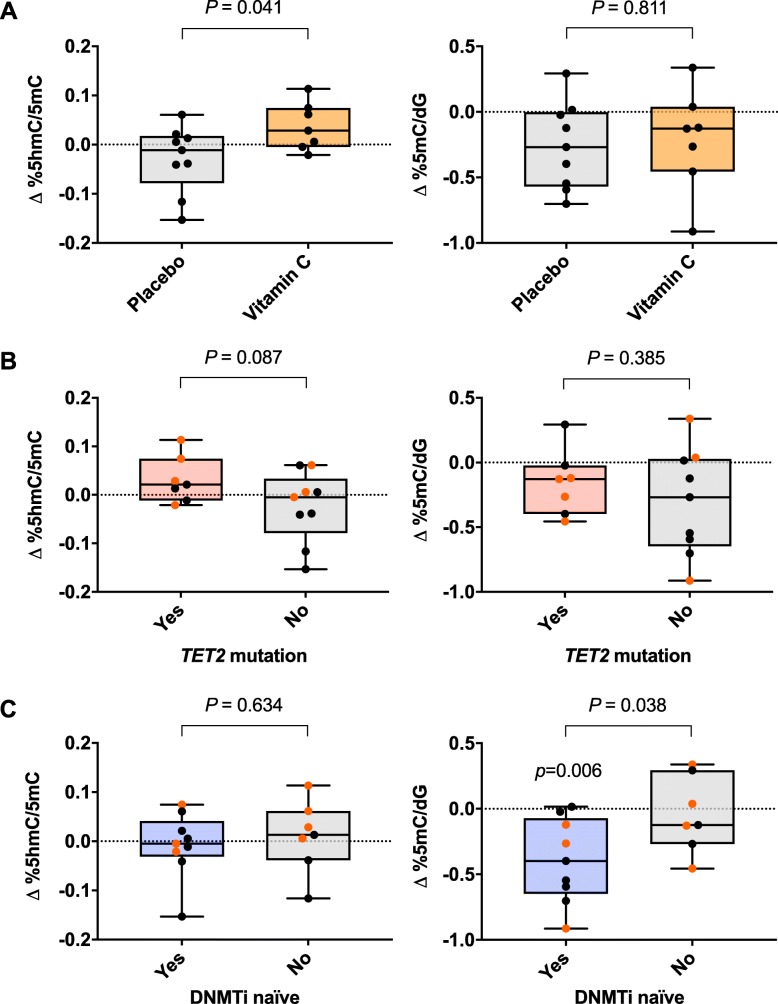
Vitamin C supplement causes increased 5hmC/5mC changes in mononuclear myeloid cells in patients treated with 5-azacytidine. Changes in the global 5hmC/5mC ratio and 5mC levels from baseline (C1D1) to end of study (C3D28) in: **a** patients receiving vitamin C supplement (500 mg, *n* = 7) or placebo (*n* = 9), **b** patients with (*n* = 7) or without (*n* = 9) *TET2* mutations, and **c** DNMTi naïve (*n* = 9) vs non-naïve (*n* = 7) patients. Difference between groups is analysed with a Welch two-sample *t* test. Percentages of global 5hmC and 5mC relative to total levels of deoxyguanosine (dG) were measured using mass spectrometry (LC–MS/MS). The 5hmC/dG level is further related to its substrate, i.e. to the 5mC/dG level (5hmC/5mC)

The potential influence of vitamin C on the activities of the TET enzymes was estimated from levels of global 5hmC in relation to 5mC (5hmC/5mC) in the patients’ blood cells. Baseline vitamin C levels were not significantly correlated with global 5hmC/5mC levels and there was no significant influence from age (Additional file 1: Table S5B). Interestingly, in patients receiving vitamin C, the change in 5hmC/5mC levels from baseline to end of the study was significantly higher than in the placebo group (0.037% vs − 0.029%, 95% CI [− 0.129, − 0.003], *P* = 0.041; Fig. 2a). This effect is independent of baseline vitamin C levels because a linear ridge regression, adjusted for baseline vitamin C levels, identified a similar significant difference in the change in 5hmC/5mC levels between the placebo and vitamin C groups (*P* = 0.031), and the estimated effect of baseline vitamin C was not significant (*P* = 0.951). Further, an interaction between supplementation and baseline vitamin C levels was not significant (*P* = 0.562) and its inclusion in the model did not affect the significance of supplementation (*P* = 0.041). These further analyses confirmed that supplementing with vitamin C results in a larger change in 5hmC/5mC relative to a placebo group, regardless of the baseline vitamin C levels in these patients. Moreover, the average change in 5hmC/5mC in patients on vitamin C supplement (0.037 ± 0.02%) was significantly greater than a mean change of 0 (*P* = 0.045). The sample size did not allow us to conclude whether the combined effect of 5-azacytidine and vitamin C on the change in 5hmC/5mC differs in patients with (*n* = 7) or without (*n* = 9) a *TET2* mutation. However, we did observe a trend towards a larger increase in 5hmC/5mC after 5-azacytidine treatment in patients with *TET2* mutations (*P* = 0.087; Fig. 2b).

### Global 5mC levels are higher in patients with severe vitamin C deficiency

**Fig. 3:**
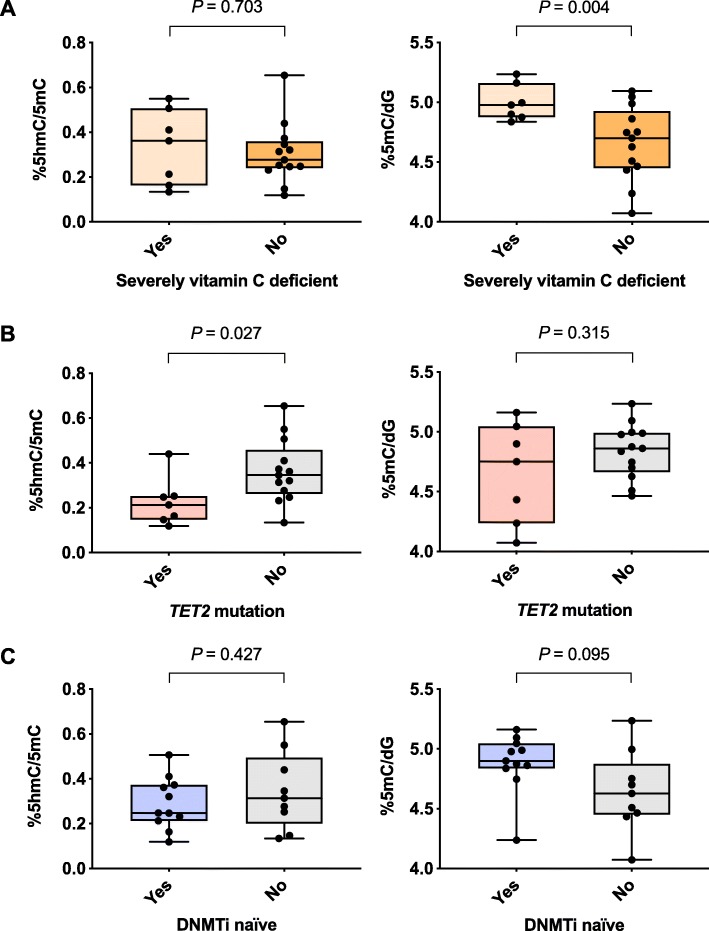
Influence of severe vitamin C deficiency (**a**), *TET2* mutations (**b**), and DNMTi naivety (**c**) on global 5hmC/5mC or 5mC levels at baseline. Global 5hmC/5mC ratio and 5mC levels in: **a** patients with (*n* = 7) vs without (*n* = 13) severe vitamin C deficiency (plasma levels < 11.4 μM) at baseline, **b** patients with (*n* = 7) vs without (*n* = 13) a *TET2* mutation, and **c** DNMTi naïve (*n* = 11) vs non-naïve (*n* = 9) patients. Difference between groups is analysed with a Welch two-sample *t* test. Global 5mC and 5hmC levels are quoted relative to total levels of deoxyguanosine (dG). The 5hmC/dG level is further related to its substrate, i.e. to the 5mC/dG level (5hmC/5mC)

Global 5mC levels were inversely associated with age (estimate ± SE, − 0.020 ± 0.008; *P* = 0.021; Additional file 1: Table S5B). Patients with severe vitamin C deficiency had significantly higher global 5mC levels (4.997 vs 4.656, 95% CI [0.126, 0.556], *P* = 0.004; Fig. 3a). At baseline, global 5hmC/5mC levels were lower in the seven patients with *TET2* mutations (0.363 vs 0.226%, 95% CI [0.018, 0.257], *P* = 0.027, Fig. 3b). As expected, the 11 patients who were DNMTi naïve had a tendency towards higher levels of 5mC at baseline (4.884 vs 4.643, 95% CI [− 0.529, 0.048], *P* = 0.095; Fig. 3c) and showed a larger reduction in 5mC levels during the study regimen compared to non-naïve patients (*P* = 0.038; Fig. 2c). Baseline global 5hmC/5mC (*P* = 0.91) or 5mC (*P* = 0.63) levels were not significantly different in patients randomized for vitamin C supplementation or placebo.

### Upregulation of viral defence genes in DNMTi naïve patients on vitamin C and 5-azacytidine combination treatment

**Fig. 4:**
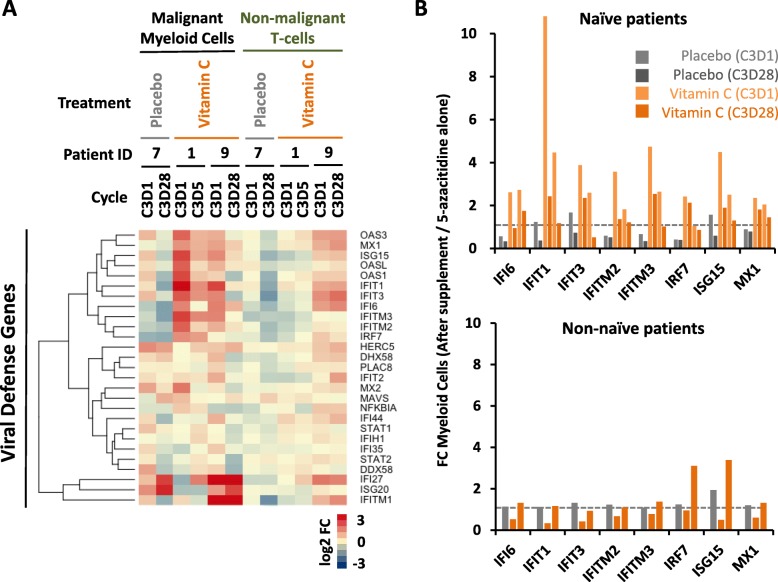
Vitamin C supplement causes upregulation of genes in the viral defence pathway in malignant myeloid cancer cells from DNMTi naïve, but not non-naïve, patients. **a** Heatmap representing the expression changes of viral defence genes in DNMTi naïve patients (patient ID 1 and 9 receiving vitamin C and patient ID 7 receiving placebo) analysed by RNAseq. **b** The effect of vitamin C on the mRNA expression of genes in the viral defence pathway is different in DNMTi naïve (top panel, *n* = 3) and DNMTi non-naïve (bottom panel, *n* = 3) patients. The top panel represents fold changes (before vs after supplement) of the viral defence genes that were upregulated in DNMTi naïve patients receiving either vitamin C supplement (patient ID 1 and 9; C3D1 vs C2D1, light orange; C3D28 vs C2D1, orange) or placebo (patient ID 7; C3D1 vs C2D1, grey; C3D28 vs C2D1, dark grey)

The differences (vitamin C vs placebo) in gene expression were analysed in six patients (four vitamin C, two placebos) by RNAseq. Compared to placebo, vitamin C-supplemented patients had increased upregulation of several viral defence genes in malignant myeloid cells (but not T cells) from patients that were DNMTi naïve (two vitamin C, 1 placebo) at study entry (Fig. 4). However, the non-naïve patients who received vitamin C did not show a similar upregulation of these genes compared to placebo.

## Discussion

We clearly show that oral supplementation can restore plasma vitamin C in patients with myeloid cancers to the levels present in the healthy population. This suggests that the deficiencies in patients with haematological malignancies are not caused by a physiological condition such as malabsorption. Moreover, we show for the first time *in patients* that global 5hmC/5mC levels increase significantly more in vitamin C-supplemented patients treated with DNMTis, and that global 5mC levels are higher in patients with severe vitamin C deficiency. The vitamin C-induced increase in global 5hmC/5mC changes in patients, plus the synergy between DNMTis and vitamin C on 5hmC conversion and increased expression of ERVs and viral defence genes that we observed in tissue culture [11], suggest that the efficacy of DNMTis might be increased by a combinatorial approach.

Interestingly, we also observe that certain viral defence genes show increased upregulation in the malignant myeloid cells from vitamin C-treated DNMTi naïve patients compared to those treated with 5-azacytidine alone. The absence of a similar upregulation by vitamin C in the non-naïve patients, or in T cells from all patients, may be due to the larger amount of dividing myeloid cells in DNMTi naïve patients that are more susceptible to vitamin C and 5-azacytidine combination treatment. Whether upregulation of viral defence genes via the combination treatment also influences the clinical responses is a subject for further investigations. Our limited data set and the short intervention period obviously do not allow for a determination of a potential beneficial clinical effect of including vitamin C in the standard treatment regimen. Therefore, we are opening a large placebo-controlled randomized clinical trial to evaluate the outcomes of patients treated with vitamin C supplementation to 5-azacytidine (NCT03999723).

A weakness of our study is that we have measured only the plasma concentration of vitamin C, while the concentrations within cells, particularly within the bone marrow, may be more relevant to the patients’ response. However, kinetics studies in healthy volunteers show that intracellular vitamin C concentrations (for example, in neutrophils) are saturated at a steady-state concentration of about 70 μM [17, 18]. This suggests that saturation of the bone marrow is achievable through oral administration, although the dose required in patients is not yet known.

It has recently been shown that vitamin C is essential for stem cell differentiation in vitamin C-deficient *Tet2-*mutated mice. Dietary vitamin C was shown to compensate for *Tet2* hetero- and homozygosity and postpone leukaemogenesis [19], whereas in vitamin C *sufficient* mice, pharmacological (high-dose intravenous) doses could compensate for the loss of Tet2 protein [20]. Both studies show that vitamin C can normalize myeloid differentiation and induce cell death [19, 20].

*In vitro* studies of solid cancers have shown beneficial effects of pharmacological doses of vitamin C by its ability to cause increased reactive oxygen species in cancer cells [21, 22]. The synergistic effect of oral vitamin C and DNMTis may act via a different mechanism, i.e. by upregulation of ERVs and the viral defence pathway, as demonstrated by our studies in cell culture [11].

## Conclusions

In conclusion, our molecular data suggest that normalization of plasma vitamin C by oral supplementation in patients treated with 5-azacytidine can increase the change in global 5hmC/5mC levels and may also upregulate a set of genes in the viral defence pathway. The induction of viral mimicry has been suggested to be positively associated with patients’ response to DNMTis. Notably, vitamin C is also a co-factor for Jumonji enzymes [6], which may have both positive and negative effects on cell proliferation. These biological observations warrant further study; however, we believe that only a large randomized, placebo-controlled clinical trial with a solid clinical endpoint can clarify whether vitamin C should be added to the standard of care of patients with myeloid cancers treated with DNMTis.

## Materials and methods

### Study design

We performed a randomized, placebo-controlled, clinical pilot study. The study was blinded for participants, care providers, and clinical investigators. After informed consent, we enrolled a total of 20 patients (9 MDS, 7 AML, and 4 CMML patients) undergoing treatment with 5-azacytidine (100 mg/m^2^ daily for the first 5 days (d) of each 28d cycle) in August 2016–March 2017 at the outpatient clinics at the Departments of Haematology at Rigshospitalet and Herlev Hospital in Copenhagen, Denmark. Inclusion criteria were patients diagnosed with MDS, AML, or CMML according to the WHO 2016 criteria who were planned for or on ongoing 5-azacytidine treatment. Exclusion criteria were other active cancer treatments within the past 6 months and ECOG performance score ≥ 3, and unwillingness to discontinue all use of vitamin C supplementation including multivitamins at inclusion. Appointment lists for the outpatient clinics were screened for eligible patients. Patients meeting the in- and exclusion criteria were offered participation at a private conversation with the study responsible medical doctor. If requested, patients were offered a 24-h cooling-off period. The patients, who had been treated with between 0 and 7 cycles of 5-azacytidine at time of inclusion, were followed for a total of 3 treatment cycles after enrolment (approx. 12 weeks, 84d; last sample collected in May 2017). Previous intake of vitamin C supplements by the participants was discontinued at the time of inclusion to the trial, i.e. 4 weeks before vitamin C or placebo supplementation was initiated. From day 1 of treatment cycle 2 (C2D1) in the trial regimen, the patients were randomized to receive oral doses of vitamin C (500 mg/d; *n* = 10) or placebo (*n* = 10). Patients were asked to take their supplement (one tablet) every evening and not to eat any fruits or vegetables on the mornings before study-related blood sampling. Blood samples were drawn immediately before 5-azacytidine was administered on day 1 and 5 of each treatment cycle and by the end of the third treatment cycle (Fig. 1a). Three patients in the vitamin C arm did not complete the study due to death (*n* = 2) or voluntary termination (*n* = 1), all between C2D1 and C3D1 in the study regimen.

### Randomization and masking

The random allocation sequence was generated in one block using randomization.com via Glostrup Pharmacy, Denmark, who also supplied the study medication. Allocation to the intervention or the placebo arm was dependent upon the chronological order of inclusion of patients as the medication containers were prelabelled with sequential study IDs. The allocation sequence was kept in a sealed envelope that was not opened until after study completion.

### Vitamin C and iron

Blood samples were immediately acidified by addition of 10% meta-phosphoric acid, which is necessary to ensure the stability of ascorbic acid [23], and the samples were subsequently analysed by high-performance liquid chromatography. Analyses of plasma iron, ferritin, and transferrin were conducted on Roche/Hitachi cobas c (iron, transferrin) and e (ferritin) systems (Roche, Mannheim, Germany; see Additional file 1: Supplementary materials and methods).

### Mutation analysis

The mutational status of the 20 most commonly mutated genes in MDS [1] were conducted by targeted next-generation sequencing of DNA from granulocytes at baseline (C1D1). We report mutations resulting in a change in amino acid sequence that was not reported as a common SNP and that had a variant allele frequency above 5%.

### DNA methylation and hydroxymethylation

Global levels of 5mC and 5hmC were measured with liquid chromatography-tandem mass spectrometry (LC–MS/MS) in DNA extracted from mononuclear myeloid cells (mononuclear cells wherefrom CD3^+^ T cells and CD19^+^ B cells had been sorted out using magnetic cell separation) and quoted relatively to total levels of deoxyguanosine (dG; see Additional file 1: Supplementary materials and methods). For one patient in the placebo arm, mass spectrometry analysis failed for the end-of-study sample. Therefore, the change in 5hmC/5mC and 5mC is based on data from 16 patients (9 in the placebo arm; 7 in the vitamin C arm).

### Gene expression analysis

Total RNAseq analysis was performed on RNA samples from mononuclear myeloid cells and T cells from six of the patients (four vitamin C, two placebos) at baseline and at the end of the study. Three of the patients were DNMTi naïve (two vitamin C, one placebo); to only evaluate the effect of vitamin C, the C2D1 sample (when the patient had been exposed to 5-azacytidine treatment alone) was used as a baseline for these patients. RNAseq was also performed on the sample taken after 1 cycle of DNMTi + vitamin C supplementation (C3D1) in DNMTi naïve patients. cDNA libraries were prepared using RNA HyperPrep Kits with RiboErase (KAPA Biosystems) according to the manufacturer’s instructions and sequencing performed on a NextSeq 500 instrument (Illumina, San Diego, CA, USA; see Additional file 1: Supplementary materials and methods).

### Statistical analysis

Linear mixed-effects models with random intercepts and false discovery rate adjusted contrasts were used to analyse plasma levels of vitamin C, ferritin, iron, and transferrin measured over time. Because some patients took a dietary supplement before study inclusion, we used the lowest vitamin C concentration from blood samples C1D1, C1D5, or C2D1 as the baseline level of vitamin C. Standard linear regression was used to assess associations between vitamin C and each of IPSS-R score, haemoglobin, blast percentage, number of blood transfusions, and WHO diagnosis; all models were adjusted for age and sex. Differences in baseline vitamin C, ferritin, iron, and transferrin were analysed via Student’s *t* test with Box-Cox transformations as needed when significant outliers (via Grubb’s test and/or Cook’s distance) or non-normality (via Shapiro-Wilks test) were detected; all assumptions were met after transformation. Differences in 5mC and 5hmC/5mC levels between groups were analysed with Welch’s two-sample *t* tests, and two additional models were used to assess the sensitivity of these results to baseline vitamin C levels. The first was a linear ridge regression fit via the R package *ridge* [24], adjusted for baseline vitamin C levels, both with and without an interaction term. The second was a weighted linear regression, with weights estimated via the genetic matching algorithm from the R package *MatchIt* [25], which seeks to non-parametrically balance the baseline vitamin C levels between the two groups. Standard linear regression models were used to test for associations between baseline levels and continuous clinical variables. Normality of residuals for all linear models was assessed visually using qq-plots; no extreme deviations were found. All hypothesis tests were two-sided, except for a *t* test to determine if the change in 5hmC/5mC was greater than 0 for the vitamin C group. The significance level for all tests was 0.05. All analyses were performed using R v3.4.4 (https://cran.r-project.org/).

## Supplementary information

**Additional file 1: Supplementary information. Supplementary materials and methods. Table S1.** Molecular and epigenetic features of the included MDS, CMML and AML patients randomized for either vitamin C or placebo supplementation. **Table S2.** Mutations in *DNMT3A, IDH2* and *TET2* in the enrolled MDS, CMML and AML patients. **Table S3.** Plasma vitamin C (vitC) concentration differentials between baseline (Pre vitC, C1D5, C2D1) and after short-term (Initial vitC, 4 d, C2D5) and longer-term (Stable vitC, 4–8 weeks, C3D1, C3D5, C3D28) supplementation of either 500 mg of vitamin C or placebo tablets. **Table S4.** Associations between plasma levels of vitamin C and plasma levels of ferritin, iron, and transferrin during each treatment cycle/treatment day (C*x*D*x*). **Table S5.** A) Associations between plasma levels of vitamin C at baseline and IPSS–R score (MDS prognostic risk category), haemoglobin, blast percentage, number of blood transfusions, and WHO diagnosis. B) Associations between baseline levels of global 5mC or 5hmC/5mC and plasma vitamin C level or age. **Fig S1.** Plasma vitamin C levels at baseline for patients with and without mutations in the DNA methylation regulators *TET2* (**A**; *n* = 7), *DNMT3A* (**B**; *n* = 5) and *IDH2* (*n* = 2). **Fig S2.** Plasma levels of iron, ferritin, and transferrin in all participants in the placebo and vitamin C arm, respectively, as a function of treatment cycle and day.

## Data Availability

The RNAseq data used and analysed during the current study will be made available upon request.

## Abbreviations

5hmC: 5-Hydroxymethylcytosine
5mC: 5-Methylcytosine
AML: Acute myeloid leukaemia
CMML: Chronic myelomonocytic leukaemia
dG: Deoxyguanosine
DNMTi: DNA methyltransferase inhibitor
ERVs: Endogenous retroviruses
IPSS-R: Revised international prognostic scoring system
MDS: Myelodysplastic syndrome
