# Supplementary material for: Oral vitamin C supplementation to patients with myeloid cancer on azacitidine treatment: Normalization of plasma vitamin C induces epigenetic changes
Source: Clin Epigenetics. 2019 Oct 17;11:143. doi: 10.1186/s13148-019-0739-5 (PMC6798470; doi:10.1186/s13148-019-0739-5)
Supplement: Supplementary file 1 — Additional file 1: Supplementary information. Supplementary materials and methods. Table S1. Molecular and epigenetic features of the included MDS, CMML and AML patients randomized for either vitamin C or placebo supplementation. Table S2. Mutations in DNMT3A, IDH2 and TET2 in the enrolled MDS, CMML and AML patients. Table S3. Plasma vitamin C (vitC) concentration differentials between baseline (Pre vitC, C1D5, C2D1) and after short-term (Initial vitC, 4 d, C2D5) and longer-term (Stable vitC, 4–8 weeks, C3D1, C3D5, C3D28) supplementation of either 500 mg of vitamin C or placebo tablets. Table S4. Associations between plasma levels of vitamin C and plasma levels of ferritin, iron, and transferrin during each treatment cycle/treatment day (CxDx). Table S5. A) Associations between plasma levels of vitamin C at baseline and IPSS–R score (MDS prognostic risk category), haemoglobin, blast percentage, number of blood transfusions, and WHO diagnosis. B) Associations between baseline levels of global 5mC or 5hmC/5mC and plasma vitamin C level or age. Fig S1. Plasma vitamin C levels at baseline for patients with and without mutations in the DNA methylation regulators TET2 (A; n = 7), DNMT3A (B; n = 5) and IDH2 (n = 2). Fig S2. Plasma levels of iron, ferritin, and transferrin in all participants in the placebo and vitamin C arm, respectively, as a function of treatment cycle and day. [file 13148_2019_739_MOESM1_ESM.docx]

**Additional file 1: Supplementary information**

**Materials and methods**

**Study design and study population**

In a double-blinded fashion, we assessed 23 Danish MDS, CMML and AML patients being treated with a DNMTi (5-azacytidine, 100 mg/m^2^ daily for the first 5 days within a 28-day cycle) for eligibility, excluded three patients who did not meet the inclusion criteria (n=2) or declined to participate (n=1) and randomly assigned 20 patients to receive either a daily tablet of 500 mg of vitamin C or placebo. All patients were followed without intervention from day 1 to day 28 of their next treatment cycle. In the following two treatment cycles (from day 28 to day 84 in the study period), patients received either 500 mg vitamin C or placebo supplement daily (1:1). Six patients were taking a multivitamin supplement at the time of inclusion; they all agreed to avoid taking that supplement during the study period. On days one and five (right before DNMTi injection) in all three treatment cycles and on day 28 of the third treatment cycle, 45 mL of peripheral blood was drawn from the median cubital vein into K_3_-EDTA tubes. Two patients in the vitamin C arm had not taken their supplement the last four days of the study regimen; the drop in vitamin C levels at C3D28 might be related to this. One other patient in the vitamin C arm was advised by his physician not to take the supplement the last 1-2 weeks of the study; the last blood sample while the patient was still on vitamin C supplementation (C3D5) was therefore used for the final 5hmC and 5mC analysis. One patient did not have enough mononuclear cells at C1D1 and the 5hmC/5mC baseline was therefore obtained at C2D1, where the vitamin C level was slightly higher (18.2 µM) than at C1D1 (9.7 µM).

**Vitamin C analysis**

Within 5 min of blood sampling, plasma samples were added to 10% *meta*-phosphoric acid (MPA) containing 2 mM Na_2_-EDTA (for vitamin C analysis), and together with crude plasma samples (for iron analysis) stored at −80 °C.^1^ Total vitamin C, i.e., ascorbate + dehydroascorbic acid (the oxidized form of vitamin C), was quantified by high-performance liquid chromatography with coulometric detection.^2^

**Ferritin, transferrin and iron analysis**

Peripheral blood was centrifuged immediately after the sample was taken and plasma was stored at −80 °C. Plasma levels of ferritin, transferrin, and free iron were analyzed at the Department of Clinical Biochemistry, Rigshospitalet, on standard equipment, i.e., Roche/Hitachi cobas c (iron, transferrin) and e (ferritin) systems (Roche, Basel, Switzerland).

**Next generation sequencing**

Samples were analyzed using a custom designed multiplex Ion Ampliseq panel (Ampliseq designer, Thermo Fischer Scientific, Waltham, MA) including the following 20 genes: *IDH1, IDH2, TET2, DNMT3A, ASXL1, TP53, NRAS, KRAS, CBL, JAK2, GATA2, CEBPA, RUNX1, SF3B1, U2AF1, SRSF2, ZRSR2, EZH2, SETBP1*, and *ETV6*. Initial DNA quantification was carried out using Qubit broad range assay. Library construction was performed by means of Ion Ampliseq technology and quantification of the final library was performed by qPCR with TaqMan Ion library quantification kit. Template preparation was automatically carried out on the Ion Chef Instrument using Hi-Q technology and reagents. Five samples, with unique barcodes, were simultaneously loaded on a 318 Ion Chip. Subsequent sequencing was carried out on the Ion PGM System using Hi-Q technology. All of the aforementioned steps were carried out according to manufacture r’s instructions and reagents and equipment manufactured by Thermo Fischer Scientific (Waltham, MA).

**Isolation of cells and nucleic acid extraction from human peripheral blood**

CD3^+^ and CD19^+^ cells were isolated from live frozen peripheral blood mononuclear cells using magnetic bead-based separation with positive selection kits for human CD3^+^ and CD19^+^ cells from StemCell Technologies according to the manufacturer’s instructions. CD3^+^ cells were further used as controls. CD3- and CD19-depleted cells were used as cells of interest to investigate mostly myeloid cells. The isolated cells were subjected to total DNA, RNA and miRNA extraction using the AllPrep DNA/RNA/miRNA Universal Kit (Qiagen) according to the manufacturer´s instructions including stringent DNase I cleanup of contaminating genomic DNA. Concentrations were measured with a Qubit 2.0 Fluorometer (Thermo Fischer Scientific).

**Mass spectrometry**

Isolated genomic DNA from myeloid cells was further purified with the Genomic DNA Clean and Concentrator kit (Zymo Research, Irvine, CA, USA) following the manufacturer’s instructions and eluted in LC/MS grade water (Thermo Fisher Scientific). Enzyme mix provided by New England Biolabs (Ipswich, MA, USA) was used to digest DNA to nucleosides. Standard nucleosides were diluted in different ratios using LC-MS water. Isotope labelled-internal standards (provided by T. Carell) were spiked with identical amounts in the different dilutions of standards and in the samples. Chromatographic separation of nucleosides was achieved using an Agilent RRHD Eclipse Plus C18 2.1 × 100 mm 1.8 μm column. The method used for the analysis using Agilent 6490 triple quadrupole mass spectrometer hyphenated to the UHPLC 1290 was previously described.^3^ A standard curve was generated by dividing unlabeled over isotope-labelled nucleosides and used to convert the peak area values to quantities. The signal-to-noise used for quantification is above 10, calculated using the peak-to-peak method. Global 5mC and 5hmC levels are quoted relative to total levels of deoxyguanosine (dG). The 5hmC/dG level is further related to its substrate, i.e. to the 5mC/dG level (5hmC/5mC).

**RNAseq**

Total RNA-seq was performed on 24 RNA samples from 6 patients. cDNA libraries were prepared using RNA HyperPrep Kits with RiboErase (KAPA Biosystems) according to the manufacturer’s instructions and sequencing was performed on a NextSeq 500 instrument (Illumina). Sequencing reads were aligned against the human GRCh38 reference genome by using the bcbio nextgen (<https://bcbio-nextgen.readthedocs.io/en/latest/>) automatic RNAseq pipeline, and hisat2 as sequence aligner. Raw read counts where subjected to post-processing using DESeq2's DESeqDataSetFromMatrix function, using treatment and batch information in the dataset for the design.

**References**

1 Lykkesfeldt J. Determination of ascorbic acid and dehydroascorbic acid in biological samples by high-performance liquid chromatography using subtraction methods: reliable reduction with tris[2-carboxyethyl]phosphine hydrochloride. *Analytical biochemistry* 2000; **282**: 89–93.

2 Lykkesfeldt J. Ascorbate and dehydroascorbic acid as reliable biomarkers of oxidative stress: analytical reproducibility and long-term stability of plasma samples subjected to acidic deproteinization. *Cancer epidemiology, biomarkers & prevention : a publication of the American Association for Cancer Research, cosponsored by the American Society of Preventive Oncology* 2007; **16**: 2513–6.

3 Leitch HG, McEwen KR, Turp A, *et al.* Naive pluripotency is associated with global DNA hypomethylation. *Nature structural & molecular biology* 2013; **20**: 311–6.

**Table S1.**

**Molecular and epigenetic features of the included MDS, CMML and AML patients randomized for either vitamin C or placebo supplementation.**

| **ID** | **Treatment** | **Sex** | **Age (yrs)** | **WHO diagnosis** | **IPSS-R or CPSS** | **Epigenetic mutations** | **Preceding DNMTi treatment (cycles)** | **Baseline vit C (µM)*** | **Baseline 5mC** | **Baseline 5hmC/5mC** | **End of study**  **vit C (µM)^#^** | **End of study 5mC** | **End of study 5hmC/5mC** |
| --- | --- | --- | --- | --- | --- | --- | --- | --- | --- | --- | --- | --- | --- |
| 1 | Vitamin C | M | 71 | MDS-EB-2 | High | *TET2* | - | 4.58 | 5.16 | 0.21 | 74.53 | 5.04 | 0.29 |
| 2 | Placebo | F | 84 | AML^†^ | Very high | *TET2* | 7 | 69.44 | 4.07 | 0.15 | 49.50 | 4.37 | 0.16 |
| 3 | Vitamin C | M | 78 | MDS-EB-1 | Intermed. | - | 3 | 5.14 | 5.00 | 0.55 | 52.09 | 5.03 | 0.56 |
| 4 | Vitamin C | M | 77 | MDS-EB-2 | High | *DNMT3A, IDH2, TET2* | 6 | 36.70 | 4.75 | 0.25 | 64.66 | 4.62 | 0.28 |
| 5 | Placebo | M | 72 | AML | - | - | - | 7.97 | 4.88 | 0.41 | 38.44 | 4.33 | 0.42 |
| 6 | Placebo | M | 72 | CMML-2^1^ | Intermed. | *TET2* | - | 27.84 | 5.05 | 0.12 | 21.74 | 4.65 | 0.11 |
| 7 | Placebo | F | 64 | AML | - | - | - | 5.90 | 4.84 | 0.51 | 4.57 | 4.24 | 0.35 |
| 8 | Placebo | F | 81 | AML^†^ | Intermed. | - | - | 31.98 | 4.86 | 0.25 | 5.70 | 4.88 | 0.31 |
| 9 | Vitamin C | M | 72 | MDS-EB-2 | Intermed. | - | - | 10.15 | 4.98 | 0.36 | 75.79 | 4.06 | 0.36 |
| 10 | Vitamin C | M | 76 | AML | Intermed. | *IDH2* | - | 14.50 | 4.75 | 0.23 | n/a | n/a | n/a |
| 11 | Vitamin C | M | 84 | MDS-EB-2 | Intermed. | *DNMT3A, TET2* | - | 18.25 | 4.24 | 0.25 | 67.06 | 3.97 | 0.23 |
| 12 | Vitamin C | M | 75 | AML | - | *TET2* | 2 | 12.70 | 4.43 | 0.44 | 62.75^2^ | 3.98^2^ | 0.55^2^ |
| 13 | Placebo | M | 76 | MDS-EB-2 | High | *DNMT3A* | 2 | 40.53 | 4.51 | 0.65 | 50.90 | 4.39 | 0.62 |
| 14 | Placebo | F | 57 | AML | - | *TET2* | - | 7.53 | 4.90 | 0.16 | 14.73 | 4.88 | 0.18 |
| 15 | Placebo | F | 58 | MDS-EB-2 | High | - | - | 14.20 | 4.99 | 0.32 | 60.40 | 4.29 | 0.28 |
| 16 | Placebo | M | 70 | MDS-EB-2 | Very high | - | 1 | 9.42 | 4.70^1^ | 0.28^3^ | 8.84 | n/a | n/a |
| 17 | Vitamin C | F | 76 | CMML-1 | High | - | 2 | 17.79 | 4.47 | 0.31 | 64.82 | 4.80 | 0.37 |
| 18 | Vitamin C | M | 76 | CMML-2 | Int-2 | - | 1 | 3.42 | 5.24 | 0.13 | n/a | n/a | n/a |
| 19 | Vitamin C | M | 70 | CMML-1 | Int-2 | *DNMT3A* | - | 15.55 | 5.09 | 0.37 | n/a | n/a | n/a |
| 20 | Placebo | M | 70 | MDS-EB-2 | High | *DNMT3A* | 1 | 43.35 | 4.63 | 0.35 | 50.11 | 4.359 | 0.23 |
| Global %5mC/dG levels and %5hmC/5mC from mononuclear myeloid cells. *Lowest plasma vitamin C concentration from blood samples C1D1, C1D5 and C2D1. ^#^Blood sample C3D28. ^†^AML with <30% blasts. ^1^MDS at date of disease onset. ^2^Blood sample C3D5 instead of C3D28. ^3^Blood sample C2D1 instead of C1D1. MDS-EB, myelodysplastic syndrome with excess blasts; AML, acute myeloid leukemia; CMML, chronic myelomonocytic leukemia; WHO diagnosis, diagnosis at date of treatment initiation; IPSS-R, Revised international prognostic scoring system (at date of disease onset); CPSS, CMML-specific prognostic scoring system (at date of disease onset). | | | | | | | | | | | | | |

**Table S2.**

**Mutations in *DNMT3A, IDH2* and *TET2* in the enrolled MDS, CMML and AML patients.**

| **Patient ID** | **Gene mutated** | **VAF %** | **Type** | **Coding** | **Locus** | **Exon** | **Variant effect** |
| --- | --- | --- | --- | --- | --- | --- | --- |
| 1 | *TET2* | 31 | p.Arg1366His | c.4097G>A | chr4:106190819 | 9 | Missense |
| 2 | *TET2* | 48 | p.Gln321fs | c.961delC | chr4:106156059 | 3 | Frameshift/Deletion |
| 4 | *DNMT3A* | 29 | p.Arg882His | c.2645G>A | chr2:25457242 | 23 | Missense |
|  | *TET2* | 11 | p.Glu1234fs | c.3700delG | chr4:106164829 | 6 | Frameshift/Deletion |
|  | *IDH2* | 30 | p.Arg140Gln | c.419G>A | chr15:90631934 | 4 | Missense |
| 6 | *TET2* | 45 | p.Tyr380fs | c.1139_1142delACTT | chr4:106156234 | 3 | Frameshift/Deletion |
|  | *TET2* | 45 | p.Pro997fs | c.2990delC | chr4:106158087 | 3 | Frameshift/Deletion |
| 10 | *IDH2* | 45 | p.Arg140Gln | c.419G>A | chr15:90631934 | 4 | Missense |
| 11 | *DNMT3A* | 7 | p.Arg882His | c.2645G>A | chr2:25457242 | 23 | Missense |
|  | *TET2* | 49 | p.Tyr867His | c.2599T>C | chr4:106157698 | 3 | Missense |
|  | *TET2* | 49 | p.Pro1723Ser | c.5167C>T | chr4:106196834 | 11 | Missense |
| 12 | *TET2* | 50 | p.Arg1366His | c.4097G>A | chr4:106190819 | 9 | Missense |
| 13 | *DNMT3A* | 49 | p.Trp330Ter | c.990G>A | chr2:25470484 | 8 | Nonsense |
| 14 | *TET2* | 49 | p.Glu227Ter | c.679G>T | chr4:106155778 | 3 | Nonsense |
|  | *TET2* | 11 | p.Gln1654Ter | c.4960C>T | chr4:106196627 | 11 | Nonsense |
| 19 | *DNMT3A* | 44 | p.Arg882His | c.2645G>A | chr2:25457242 | 23 | Missense |
| 20 | *DNMT3A* | 8 | p.Asp529Asn | c.1585G>A | chr2:25467491 | 14 | Missense |

**Table S3.**

**Plasma vitamin C (vitC) concentration differentials between baseline (Pre vitC, C1D5, C2D1) and after short-term (Initial vitC, 4 d, C2D5) and longer-term (Stable vitC, 4-8 weeks, C3D1, C3D5, C3D28) supplementation of either 500 mg of vitamin C or placebo tablets.**

| **Contrast** | **Mean difference** | **SE** | **t-stat** | ***P*** |
| --- | --- | --- | --- | --- |
| **Placebo (n = 10)** |  |  |  |  |
| Initial vitC - Pre vitC | –1.79 | 4.10 | –0.44 | 0.88 |
| Stable vitC - Pre vitC | 0.54 | 3.46 | 0.16 | 0.88 |
| Stable vitC - Initial vitC | 2.34 | 4.19 | 0.56 | 0.88 |
| **Vitamin C (n = 10)** |  |  |  |  |
| Initial vitC - Pre vitC | 36.31 | 9.67 | 3.76 | 0.0011 |
| Stable vitC - Pre vitC | 34.85 | 7.94 | 4.39 | 0.0004 |
| Stable vitC - Initial vitC | –1.46 | 10.14 | -0.14 | 0.8866 |
| **Phase** | **Mean difference** | **SE** | **t-stat** | ***P*** |
| **Vitamin C vs Placebo** |  |  |  |  |
| Pre vitC | –0.77 | 8.49 | –0.09 | 0.9283 |
| Initial vitC | 36.07 | 10.69 | 3.37 | 0.0016 |
| Stable vitC | 32.78 | 9.08 | 3.61 | 0.0013 |

| **Table S4.**  **Associations between plasma levels of vitamin C and plasma levels of ferritin, iron, and transferrin during each treatment cycle/treatment day (C*x*D*x*).** | | | | | | |
| --- | --- | --- | --- | --- | --- | --- |
| **Treatment cycle/day** | **Regression coefficient** | **SE** | **t-stat** | ***P*** | **R2** | **FDRq*** |
| **Ferritin vs vitamin C** |  |  |  |  |  |  |
| C1D1 | –0.003 | 0.003 | –0.99 | 0.34 | 0.16 | 1 |
| C1D5 | –0.002 | 0.002 | –0.89 | 0.39 | 0.07 | 1 |
| C2D1 | –0.001 | 0.004 | –0.31 | 0.76 | 0.21 | 1 |
| C2D5 | 0.002 | 0.003 | 0.61 | 0.55 | 0.27 | 1 |
| C3D1 | 0.011 | 0.004 | 2.54 | 0.03 | 0.41 | 0.18 |
| C3D5 | 0.007 | 0.005 | 1.41 | 0.19 | 0.38 | 1 |
| C3D28 | 0.001 | 0.004 | 0.28 | 0.79 | 0.18 | 1 |
| **Iron vs vitamin C** |  |  |  |  |  |  |
| C1D1 | –1.196 | 0.617 | –1.94 | 0.07 | 0.26 | 0.49 |
| C1D5 | –0.168 | 0.479 | –0.35 | 0.73 | 0.03 | 1 |
| C2D1 | –0.866 | 0.576 | –1.50 | 0.15 | 0.30 | 0.92 |
| C2D5 | 0.245 | 0.576 | 0.43 | 0.68 | 0.26 | 1 |
| C3D1 | 1.350 | 1.080 | 1.25 | 0.24 | 0.23 | 1 |
| C3D5 | 0.557 | 0.915 | 0.61 | 0.56 | 0.30 | 1 |
| C3D28 | 0.527 | 0.732 | 0.72 | 0.49 | 0.20 | 1 |
| **Transferrin vs vitamin C** |  |  |  |  |  |  |
| C1D1 | 0.062 | 0.143 | 0.44 | 0.67 | 0.12 | 1 |
| C1D5 | 0.089 | 0.084 | 1.06 | 0.31 | 0.09 | 1 |
| C2D1 | 0.107 | 0.154 | 0.69 | 0.50 | 0.23 | 1 |
| C2D5 | 0.065 | 0.127 | 0.51 | 0.62 | 0.26 | 1 |
| C3D1 | –0.298 | 0.189 | –1.58 | 0.14 | 0.27 | 0.99 |
| C3D5 | –0.237 | 0.229 | –1.03 | 0.32 | 0.34 | 1 |
| C3D28 | –0.036 | 0.145 | –0.25 | 0.81 | 0.18 | 1 |

*False discovery rate adjusted *P* - value

**Table S5.**

**A) Associations between plasma levels of vitamin C at baseline and IPSS–R score (MDS prognostic risk category), hemoglobin, blast percentage, number of blood transfusions, and WHO diagnosis.**

| **Variable** | **Regression coefficient** | **SE** | **t–stat** | ***P*** | **R2** |
| --- | --- | --- | --- | --- | --- |
| IPSS–R score | 0.017 | 0.018 | 0.98 | 0.35 | 0.22 |
| Hemoglobin | 0.014 | 0.018 | 0.76 | 0.46 | 0.06 |
| Bone marrow blast percentage | –0.229 | 0.369 | –0.62 | 0.55 | 0.02 |
| No. of blood transfusions in the study period | –0.180 | 0.147 | –1.22 | 0.24 | 0.09 |
|  | **Mean difference** | **SE** | **t–stat** | ***P*** |  |
| WHO diagnosis (WHO)   - AML – CMML - AML – MDS - CMML – MDS | 10.21  –4.87  –15.07 | 11.34  9.48  10.43 | 0.90  –0.51  –1.45 | 0.65  0.87  0.34 |  |

IPSS-R, Revised international prognostic scoring system (at date of diagnosis for MDS and AML patients <30% bone marrow blasts). Baseline vitamin C level is a covariate in all analyses. All regression analyses are adjusted for age and sex.

**B) Associations between baseline levels of global 5mC or 5hmC/5mC and plasma vitamin C level or age.**

| **Contrast** | **Regression coefficient** | **SE** | **t-stat** | ***P*** |
| --- | --- | --- | --- | --- |
| **%5hmC/5mC (n = 20)** |  |  |  |  |
| Vitamin C | -0.0000335 | 0.000067 | -0.499 | 0.624 |
| Age | -0.000065 | 0.00021 | -0.317 | 0.755 |
| **%5mC (n = 20)** |  |  |  |  |
| Vitamin C | -0.0053 | 0.0028 | -1.901 | 0.0735 |
| Age | -0.0202 | 0.008 | -2.522 | 0.0213 |

Global 5mC and 5hmC levels are quoted relative to total levels of deoxyguanosine (dG). The 5hmC/dG level is further related to its substrate, i.e. to the 5mC/dG level (5hmC/5mC).

**Figure S1.**

Plasma vitamin C levels at baseline for patients with and without mutations in the DNA methylation regulators *TET2* (**A**; n=7), *DNMT3A* (**B**; n=5) and *IDH2* (n=2). In **B**, the pink dot represents a patient with both *DNMT3A* and *TET2* mutations, the green dot a patient with an *IDH2* mutation and the white dot a patient with *DNMT3A, IDH2* and *TET2* mutations.


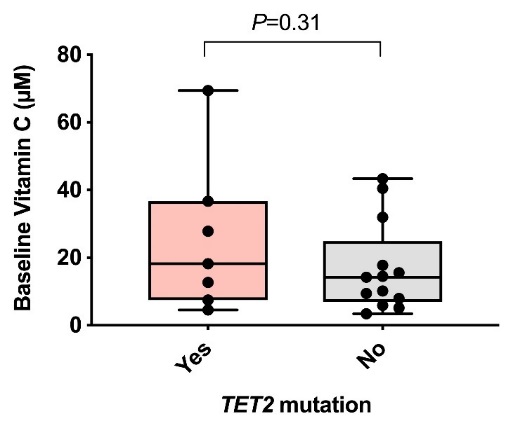


**A**


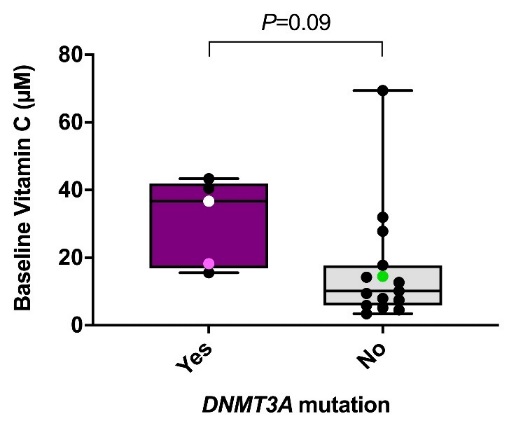


**B**

**Figure S2.**

**Plasma levels of iron, ferritin, and transferrin in all participants in the placebo and vitamin C arm, respectively, as a function of treatment cycle and day.**

Black lines indicate the low/high threshold for normal levels of ferritin (**A**; 12 to 300 ng/mL for males, 12 to 150 µg/mL for females), transferrin (**B**; 170 to 370 mg/dl) and iron (**C**; 11–32 µmol/L). Data presented as spline mean with SE ribbons for cycles; C = cycle, D = day in cycle.


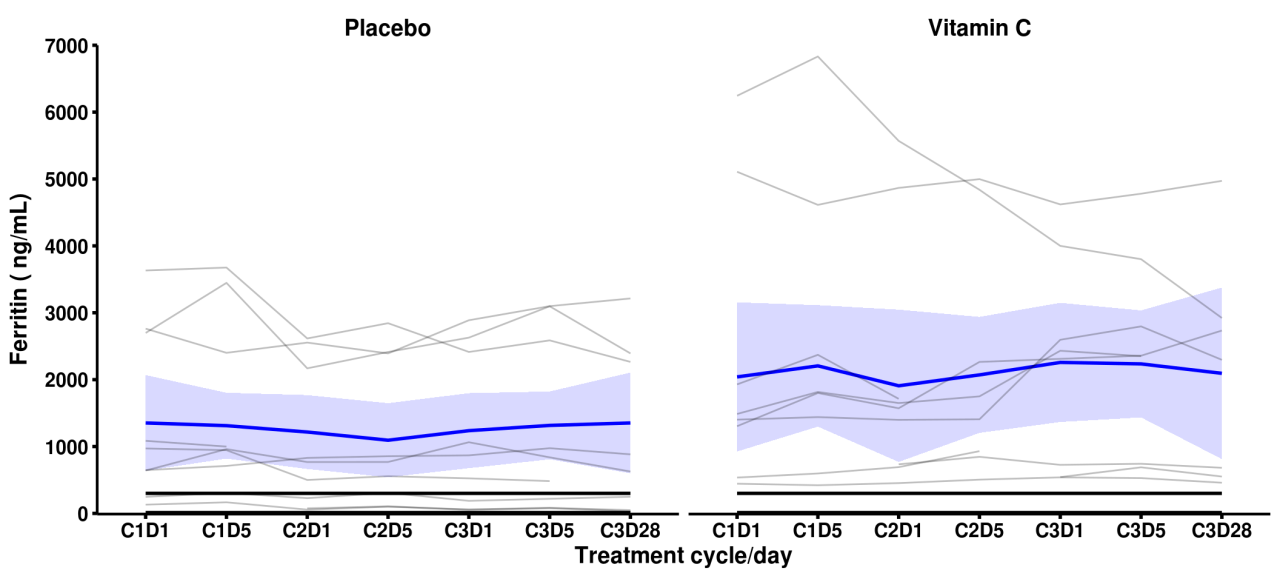


**A**

**
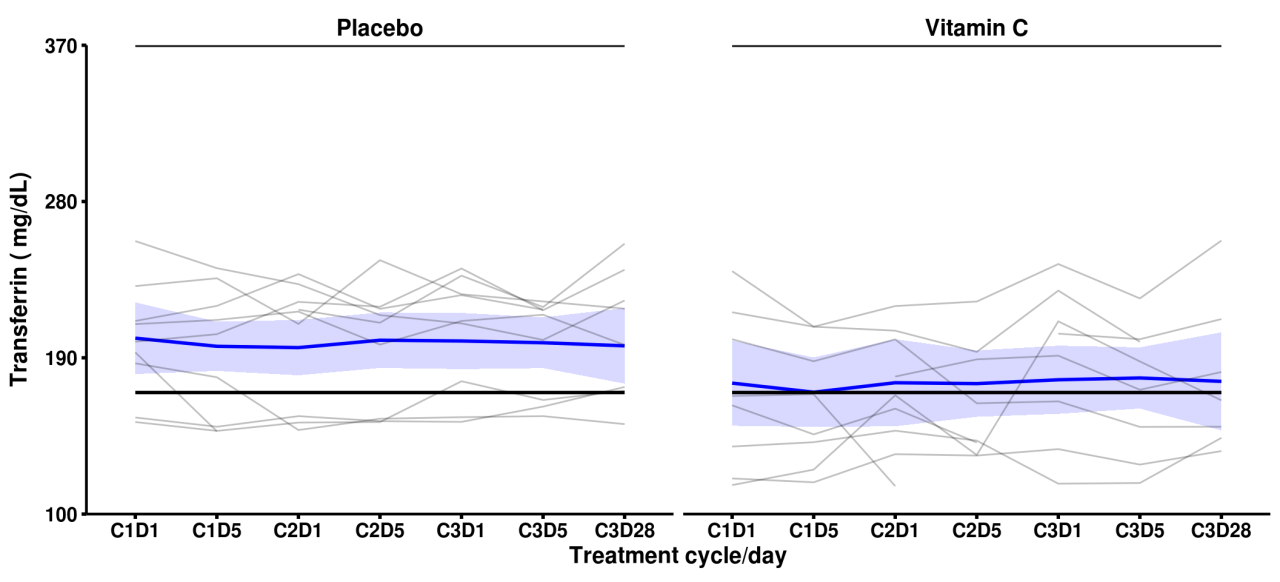
**

**B**


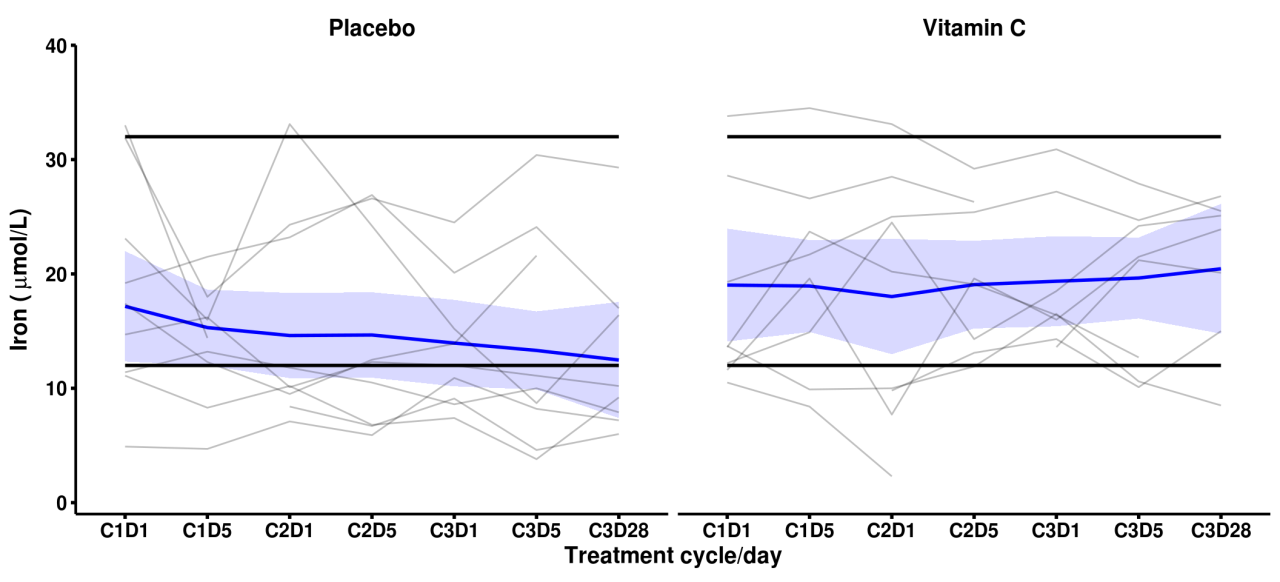


**C**
